# Risk Perceptions of Wastewater Use for Urban Agriculture in Accra, Ghana

**DOI:** 10.1371/journal.pone.0150603

**Published:** 2016-03-15

**Authors:** Prince Antwi-Agyei, Anne Peasey, Adam Biran, Jane Bruce, Jeroen Ensink

**Affiliations:** 1 Faculty of Infectious and Tropical Diseases, Environmental Health Group, London School of Hygiene and Tropical Medicine, Keppel street, WC1E 7HT, London, United Kingdom; 2 Department of Epidemiology and Public Health, University College London, 1–19 Torrington Place, WC1E 6BT, London, United Kingdom; 3 Faculty of Infectious and Tropical Diseases, London School of Hygiene and Tropical Medicine, Keppel street, WC1E 7HT, London, United Kingdom; Universidade de Brasilia, BRAZIL

## Abstract

Poor food hygiene is a significant risk to public health globally, but especially in low and middle-income countries where access to sanitation, and general hygiene remain poor. Food hygiene becomes even more pertinent when untreated, or poorly treated wastewater is used in agriculture. In such circumstances the WHO recommends the adoption of a multiple-barrier approach that prescribes health protective measures at different entry points along the food chain. This study sought to assess the knowledge and awareness of wastewater use for crop production, its related health risks, and adoption of health protective measures by farmers, market salespersons and consumers using questionnaires and focus group discussions. In the period from September 2012 to August 2013, 490 respondents were interviewed during two cropping seasons. The study found that awareness of the source of irrigation water was low among consumers and street food vendors, though higher among market vendors. In contrast, health risk awareness was generally high among salespersons and consumers, but low among farmers. The study found that consumers did not prioritize health indicators when buying produce from vendors but were motivated to buy produce, or prepared food based on taste, friendship, cost, convenience and freshness of produce. Similarly, farmers’ awareness of health risk did not influence their adoption of safer farm practices. The study recommends the promotion of interventions that would result in more direct benefits to both producers and vendors, together with hygiene education and enforcement of food safety byelaws in order to influence behaviour change, and increase the uptake of the multiple-barrier approach.

## Introduction

Globally, diarrhoea remains one of the leading causes of death in both adults and children [1]. The risk factors for diarrhoeal diseases are multi-faceted, but an estimated 94% of cases are attributed to environmental factors which include unsafe drinking water, poor sanitation and hygiene [2]. For example, an estimated 502,000 and 280,000 deaths in 2012 were associated with inadequate water and sanitation respectively from a total of 1.5 million diarrhoea related deaths [3].

The role of improved food hygiene in the transmission of diarrhoeal disease is under- researched. Food hygiene interventions, especially for food that can be eaten uncooked (e.g. salads) remain a challenge, especially in low and middle-income countries (LMICs) where access to sanitation, and general hygiene are poor [4, 5]. Food hygiene interventions are further complicated in countries where water is scarce, and wastewater is used for agricultural production [4]. The use of wastewater (defined in this study as domestic effluent of blackwater and greywater, water from commercial establishments and institutions and storm water and other run-off) in agriculture has been associated with diarrhoeal disease, and helminth infections in both farmers and consumers [6]. However, post-harvest contamination has shown to pose an even greater health risks than the use of untreated wastewater in some instances [7, 8].

To safeguard human health when wastewater is used in agriculture, the World Health Organisation (WHO) has developed guidelines to regulate the use of wastewater. The 2006 revision of the WHO guidelines recommends a multiple-barrier approach to protect consumer and farmer health [4]. The multiple-barrier approach stipulates health protection measures at different entry points along the food chain, and is particularly recommended for countries where wastewater is used without treatment [4]. Currently, the implementation and uptake of the proposed non-wastewater treatment protective measures has been slow due to attitudes and perceptions of farmers, retailers and consumers [9]. In addition, there is inadequate field-based evidence on the effectiveness and efficacy of these risk reduction measures especially regarding on-farm measures, and hygienic food marketing and food preparation at markets, homes and kitchens in low and middle-income countries [10]. Studies have suggested that interventions are more likely to be successful when they are designed to incorporate the target groups’ perceptions, attitudes, suggestions/knowledge and constraints [11, 12]. Education and awareness creation on the health risks of wastewater irrigation has been recommended as one of the health protective measures [10, 13] but prior to undertaking this, it is important to understand the diverse food safety issues and perceptions relevant to producers and consumers [9]. Health risk perceptions, describe the subjective judgement of people to health risk, or behaviours and could be triggered by factors such as tradition, family pressure, community norms, time pressure and inconvenience [9, 14]. This study sought to assess how farmers, crop handlers and consumers’ knowledge and awareness of health risks of produce irrigated by wastewater influence their buying, consumption, and adoption of food hygiene practices.

## Method

The study adopted a mixed method approach including the use of semi-structured interviews, and focus group discussions (FGDs). A total of 490 respondents, including wastewater farmers, market salespersons, street food vendors and public and domestic consumers of salad produce were interviewed from October 2012 to December 2012 (dry season), and from June to August 2013 (wet season).

### Study sites

There are an estimated 160 hectares, over seven major sites of farmland, irrigated by wastewater in Accra, Ghana. Each of the major sites has between 60 and 200 agricultural workers. The predominant sources of irrigation water at these sites are municipal wastewater sources including: open drain water, and dug-outs (ponds). Farmers apply water to their crops, which include: lettuce, cabbage, spring onions, and a host of other local vegetables using watering cans. Produce from these farms is sold to market vendors, street food vendors, and restaurants within Accra. For markets, the study focused on central markets, which have the largest population of vendors and customers, and serve as wholesale distribution centres for traders dealing in salad vegetables [15]. Unlike restaurants and hotels, the street food sector in Accra is largely informal, and hence difficult to regulate. An increasingly popular category of street food vendors in Accra is the “check-check” (fast food) seller. These vendors are found in open spaces with decorated and stylishly mounted kiosks that sell fast food especially in the evenings. Most of these food vendors have no permit, or hygiene certificates from the public health departments [16]. It must be noted that produce sold at markets and also those used to prepare salad at street food stalls, restaurants and hotels originate from different sources including wastewater fields in Accra and also from farms outside Accra, including those from other countries. This study therefore makes the assumption that not all the produce are wastewater irrigated, especially produce sourced from outside Accra which could be irrigated with safe sources of water. A map of the various study sites has been presented as a supporting information to this paper (S1 and S2 Figs).

### Sample size and selection

The sample size and selection strategy for each of the at-risk groups have been presented in Table 1. For a faecal exposure assessment study, 80 farmers were randomly selected for observation, and later interviewed [17]. Similarly, 80 market salespersons from three central markets that sold salad crops, in particularly lettuce and cabbage were randomly selected; using their market stalls, or sheds numbers for selection [18]. Sample size for consumers of salad produce was determined based on 80% power and 5% significance level to detect a 20% difference in increased awareness or knowledge of health risk associated wastewater irrigation between salad consumers and non-consumers based on a similar study in Ghana [19]. This then resulted in 160 each of street food consumers and buyers of salad produce at markets (domestic consumers). Consumers of salad vegetables were randomly (systematically) selected when they bought their food, or raw produce within the observation period at the selected sites [20]. Food vendors in the selected communities in Accra could not be easily identified as they were not registered. Consequently, the 160 street food consumers were divided over 30 street food vendors (“check-check” sellers) who were randomly recruited from an already generated numbered list of food vendor stalls previously identified by a transect walk with community leaders in two communities. One community was a planned settlement (Alajo), while the other was a squatter settlement (Old Fadama).

**Table 1:** Sample size and selection strategy for at-risk groups.

At-risk group	Sample size	Selection strategy	Type of produce cultivated, sold or bought	Origin of produce or prepared salad
Farmers	80	Systematic sampling	Lettuce	Wastewater irrigated fields in Accra
Market vendors	80	Systematic sampling	Lettuce and cabbage	Farms in Accra and outside Accra (produce could be wastewater irrigated or not)
Produce buyers (domestic consumers)	160	Systematic sampling	Lettuce and cabbage	Markets in Accra (produce could be wastewater irrigated or not)
Street food vendors	30	Random sampling	Prepared salad	Farms and markets in Accra (produce could be wastewater irrigated or not)
Street food consumers	160	Systematic sampling	Prepared salad	Street food vendors in Accra (produce could be wastewater irrigated or not)
Chefs at hotels and restaurants	20	Purposive sampling	Prepared salad	Farms (Accra and outside), markets, and supermarkets in Accra. Produce could be wastewater irrigated or not

Restaurants and hotels in Accra where salad was served to the public were also included in the study during the rainy season, and were selected on the basis of their “star” rating, location and popularity. The selection was done in collaboration with the Ghana Food and Drugs Authority (FDA) from their database of restaurants and hotels.

### Data collection

#### Questionnaire

Questionnaires were verbally administered to street food vendors, and consumers of fast food between 18:00 and 21:00, while domestic consumers, farmers and market vendors were interviewed from 7:00 to 10:00 (S1 Appendix). Questionnaires covered both closed and open ended questions and were administered to respondents immediately after any observation was carried out. With farmers, questions dealt with defaecation practices, and food hygiene practices, while market and street food vendors were interviewed about awareness of the source of irrigation water or produce, health risks associated with wastewater irrigation, and whether this awareness, or the source of irrigation water influenced their buying and consumption of salad vegetables. Interviews with chefs took place at hotels and restaurants, and covered among other topics, the sources of produce for salads, and methods of treating salad. Only the chef who prepared the salad at the time of sample collection was interviewed. Questionnaires for street food, and domestic consumers included salad consumption frequency, factors that influenced their purchase and health risk awareness associated with wastewater use for irrigation.

#### Focus group discussion

Three FGDs (6 per group) were held each with market vendors and farmers who were not included in previous interviews, or observations. Focus group discussions explored similar topics as the questionnaires, and were meant to provide complementary information. Where appropriate, participants for FGDs were selected to ensure a similar sample to those questioned, or observed on the basis of gender, years of working experience, or religion. There were three researchers for each FGD, the lead conductor and two note takers. All FGDs were audio recorded and later transcribed.

### Data Analysis

All data were analysed using STATA 12 (StataCorp LP, College Station, USA). In this study, health risk perception covered awareness of the sources of irrigation water or produce, awareness/knowledge of wastewater related health risk/disease, factors that influenced consumers to buy produce or prepared food, and the adoption of risk reduction measures. The association between respondents’ knowledge and awareness of wastewater use, health risk, and consequently buying and consumption of salad, together with the adoption of health protective measures was assessed using Pearson Chi-square test, and logistic regression models. Awareness of health risk and buying, or consumption of salad were the main outcomes while respondents’ personal characteristics, awareness of source of irrigation water/health risk, or source of produce were the main exposures. Awareness of health risk meant respondents had heard that wastewater use could result in disease outcome, while awareness of the source of irrigation water meant that respondents had been told, or claim to know about the various types of water sources (taps/piped water, drain water, ponds) used to irrigate crops. Awareness of the source of irrigation water required only a “Yes” or “No” answer, and respondents were not asked to name the source of water as evidence of their reported knowledge. Findings from FGDs with market vendors, however, provided supplementary information on vendors’ awareness of irrigation water sources. However, awareness of health risk was confirmed as knowledge of the risk if respondents were able to mention correctly a disease associated with the consumption of wastewater irrigated produce. Awareness was subjectively classified as “high” if more than 50% of the specific group of respondents reported to be aware of that parameter; and “low” if those who claimed awareness was less than 50%. The same level of awareness classification was used in the FGDs, or was classified as “high” if there was a general consensus among the FGDs participants. Odds ratios (OR) were used to measure the association between exposures and binary outcomes in both univariable and multivariable logistic regression models. Only factors that were significant at 10% in the univariable analysis were included in the multivariable logistic model. Statistically significant associations between exposures and outcomes in the multivariable analysis were measured at 5% significance level using the likelihood ratio test. Data from FGDs were transcribed verbatim using Microsoft office word, and thematic content coding was used to analyse the data from predetermined themes [21]. Interaction of individual responses among the groups was taken into account and group was used as the unit of analysis. Similar to the questionnaire, awareness of risk, or the source of irrigation water and produce was defined as good if there was a general consensus among participants, or if more than half of the participants in the focus group claimed to be aware of that parameter.

### Ethical Approval

Ethical approval was received from the Ethical Review committees of the London School of Hygiene and Tropical Medicine (LSHTM, reference number—6236) in the UK and from the Noguchi Memorial Institute of Medical Research in Ghana (Reference number–DF22). The study was also explained and agreed to by local leaders. Written informed consent was then obtained from each individual respondent (counter-signed and dated by researcher) for questionnaires and FGDs after the objectives of the study and the content of the consent form have been explained to them. In very few cases, verbal consent was obtained from respondents who were unable to append their signatures on the consent forms and where no other substantive witness or volunteer was available to sign on their behalf. In such cases and as recommended by the ethics committee, the researcher signed the form indicating that all appropriate information has been explained to the respondent and that the respondent was satisfied and provided a verbal consent. This was also explained to the local leaders. The information sheets containing the contacts of the researcher and the ethic committees were also handed over to respondents if they required further information. There were no minors in this study. All respondents were assured of confidentiality and security of the information they provide and that only alphanumeric symbols were used to identify respondents. Field permit was granted by the London School of Hygiene and Tropical Medicine. The Ghana Food and Drugs Authority (FDA) granted field access to all hotels and restaurants while local government leaders at the communities (Assemblymen) approved of access to street food vendors in their communities. Access to markets and farm sites were also approved by the Secretaries and Market Queens of the market associations and the leaders of the farmer associations respectively. The field work did not involve any endangered or protective species.

## Results

### Characteristics of study respondents

The majority (95%) of farmers were males with an average age of 40 years. Almost a third (31%) of farmers had no formal education, and urban agriculture provided the main source of income for nearly 80% of farmers. Market salespersons, in contrast, were predominantly female (95%) with nearly 90% above 30 years of age. Street food vendors were mostly males (83%), had some formal education (97%), and the large majority (76%) was younger than 30 years of age. The consumers of “check-check” food, were 61% males with the majority (87%) below 30 years. Buyers of produce at markets were mostly female (84%) with an average age of 37 years.

### Awareness of sources of irrigation water and produce

**Fig 1:**
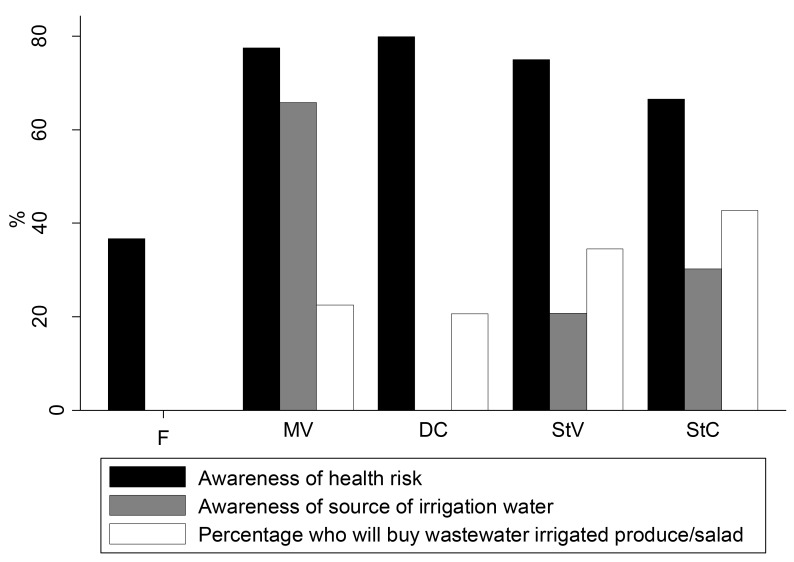
Wastewater irrigation and health risk awareness and perceptions.

Among all respondents, awareness of the source of irrigation water used by farmers for vegetable cultivation was highest among market vendors (66%, Fig 1). Market vendors also stated that produce was more likely to be irrigated with wastewater if it was cultivated within Accra than outside Accra–*“Yes*, *we are aware of the irrigation water farmers use and some customers will even ask you where you get your lettuce from*. *Some will not buy if you tell them the lettuces are from Accra”* (FGD). Contrary to market vendors, a far lower proportion of street food vendors (21%) and consumers of street food (30%) claimed being aware of the sources of irrigation water.

The questionnaire also showed that farmers in Accra were the biggest suppliers of lettuce at the markets (40%), and that more than a third (38%) of vendors bought their vegetables directly from farm gates in Accra, while the rest bought from wholesale, and retail markets within, and outside Accra. Market vendors in FGDs also claimed good or high awareness of the origin of produce: *“We know of the source of water farmers used to irrigate vegetables in Accra–you can even smell it when you buy the produce*”. At markets, less than half of domestic consumers (44%) reported being aware of the source of produce they bought at markets (Table 2). All street food vendors reported buying their produce from wholesale, or retail markets, though observations showed that some vendors bought directly from wastewater irrigated farms. Chefs at restaurants, and hotels mostly bought salad produce directly from farms, or third party suppliers (55%); with the rest buying from wholesale/retail markets (35%), or supermarkets (10%).

**Table 2:** Determinants of awareness of wastewater irrigation health risk among domestic consumers of salad (Univariable and Multivariable logistic regression models). OR^c^ = crude odds ratio, OR^a^ = adjusted odds ratio, NA = not applicable

			Univariable analysis		Multivariable analysis	
Exposure	N = 159	Awareness of risk (%)	OR^c^ (95% CI)	P* - value	OR^a^ (95% CI)	P^†^ - value
Awareness of source of produce						
No	89	70	1.0	< 0.001	1.0	0.002
Yes	69	93	5.6 (2.02, 15.40)		5.1 (1.82, 14.16)	
Religion						
Muslim	27	63	1.0	0.02	1.0	0.08
Christian	132	83	2.9 (1.19, 7.27)		2.3 (0.91, 6.01)	
Gender						
Female	135	80	1.0	0.93	NA	NA
Male	24	79	0.9 (0.33, 2.77)			
Age group						
≤ 30	57	79	1.0	0.50	NA	NA
31–40	49	84	1.4 (0.52, 3.76)			
41–50	37	73	0.7 (0.28, 1.94)			
> 50	17	88	2.1 (0.41, 10.21)			
Occupation						
Public servant	17	76	1.0	0.70	NA	NA
Traders	81	79	1.2 (0.33, 4.01)			
Vocational	49	80	1.2 (0.32, 4.49)			
Others	12	92	3.4 (0.33, 34.92)			

*P-value from logistic regression.

^†^ p–value calculated from likelihood ratio test

### Knowledge of health risks associated with wastewater irrigation

Among all study respondents, awareness of health risk associated with wastewater irrigation was highest among domestic consumers (80%) of salad vegetables, while farmers were the least (37%) likely to associate wastewater use to health risks (Fig 1)—*“I don’t think it is possible to get any disease after consuming produce irrigated with drain water*. *We even eat some of the raw lettuce on the farm*, *and we don’t always wash them*, *let alone use disinfectant” (FGD)*. Farmers claimed to wear boots to avoid cuts rather than to prevent contact to contaminated soil, or wastewater. “*We are used to walking barefoot and we don’t think there are any health effects with that*. *Farmers wear boots to protect themselves from cuts from broken bottles or other sharp materials*. *Farmers who are at sections of the farm where these sharp materials are normally wear the boots”* (FGD). Farmers’ age, sex and religion had no significant association with wastewater risk awareness. The only factor associated with farmers’ awareness that wastewater irrigation carried health risks was higher education levels, with farmers with primary education, or secondary education almost 5 and 8 times as likely to associate health risks to wastewater use, as farmers without formal education (p = 0.05). Rather than health risks, farmers were more concerned about getting support from government in terms of provision of seeds, subsidies for fertilisers and other agro-chemicals and provision of land for farming or assurance of land security. *“Government should take charge and subsidize the cost of fertilizers and vegetable seeds*, *since these are sold at a far higher price on the private market*. *It even becomes difficult to get supply at certain times” (FGD)*. Farmers in an FGD also attributed the above problems to the weak relationship that exists between local authorities and farmer associations.

Among market vendors, being aware of the source of irrigation water was associated with higher awareness of health risk (OR = 4.6, p = 0.06), though this association was non-significant (OR = 4.7, p = 0.12) when controlled for gender, age, education and religion. For domestic consumers, awareness of the source of produce was significantly associated with a higher awareness of wastewater related health risk after controlling for religion (OR = 5.1, 95% CI = 1.8–14, p = 0.002, Table 2). Gender, age and occupation of domestic consumers had no significant association on their awareness of health risk.

The majority (75%) of street food vendors also associated the consumption of wastewater irrigated produce with health risks. No association was found between risk awareness and vendor characteristics. Similar to vendors, awareness of wastewater health risks was also high among street food consumers. After controlling for religion and awareness of the source of irrigation water, male consumers of street food were almost three times (95% CI: 1.19–5.15, p = 0.02) as likely to be aware of wastewater health risk as female consumers (Table 3).

**Table 3:** Determinants of awareness of wastewater irrigation health risk among street food consumers of salad (Univariable and Multivariable logistic regression models). OR^c^ = crude odds ratio, OR^a^ = adjusted odds ratio, NA = not applicable.

			Univariable analysis		Multivariable analysis	
Exposure	N = 158	Awareness of risk (%)	OR^c^ (95% CI)	P*– value	OR^a^(95% CI)	P^†^-value
Religion						
Muslim	101	55	1.0	< 0.001	1.0	0.001
Christian	56	88	5.9 (2.42, 14.16)		4.8 (1.96, 12.02)	
Gender						
Female	63	54	1.0	0.007	1.0	0.02
Male	95	75	2.5 (1.28, 4.97)		2.8 (1.19, 5.15)	
Age group						
≤ 20	46	65	1.0	0.84	NA	NA
21–30	91	68	1.1 (0.54, 2.41)			
> 30	21	62	0.9 (0.30, 2.53)			
Occupation						
Traders	56	38	1.0	0.38	NA	NA
Student	37	22	2.2 (0.84, 5.63)			
Vocational	15	27	1.7 (0.47, 5.85)			
Scrap dealer	22	41	0.9 (0.32, 2.37)			
Other	28	39	0.9 (0.37, 2.35)			
Awareness of source of irrigation water						
No	110	60	1.0	0.09	1.0	0.05
Yes	47	81	2.8 (1.24, 6.40)		2.4 (1.0, 5.77)	

*P-value from logistic regression.

^†^ p–value calculated from likelihood ratio test

Similarly, consumers’ awareness of the source of irrigation water used for vegetable cultivation was associated with a higher awareness of health risk. Age and occupation of street food consumers had no significant influence on their awareness of wastewater health risk.

In terms of knowledge of diseases, just over 50% of all respondents did not associate any health risks with exposure to wastewater, or failed to correctly mention a disease associated with exposure to wastewater (Table 4). Knowledge of wastewater related diseases was highest among domestic consumers with 66% correctly identifying diseases such as diarrhoea/cholera and worm infections, but lowest among farmers (26%). Although awareness of wastewater health risks was high among street food consumers, it did not necessarily translate into knowledge of wastewater use related diseases, with 35% of those who were aware of the health risks unable to correctly mention a disease associated with exposure to wastewater.

**Table 4:** Proportion of respondents who mentioned diseases associated with exposure to wastewater irrigation.

Main disease mentioned	Farmers (N = 80)	Market vendors (N = 40)	St. food vendors (N = 29)	Produce buyers (N = 159)	St. food consumers (N = 158)	All respondents* (N = 466)
No risks	64%	23%	31%	20%	33%	33%
Those aware of health risks						
Diarrhoea	19%	35%	24%	41%	21%	29%
Cholera	5%	18%	24%	23%	17%	17%
Worm infection	2.5%	2%	7%	2%	5%	3.4%
Non-related ones	3.7%	15%	14%	8.8%	8%	8.4%
Cannot tell	6%	7%	0%	5.0%	16%	9%

*All respondents included farmers, market vendors, street food vendors, produce buyers and street food consumers

### Factors influencing consumers to buy produce or prepared salad food

**Fig 2:**
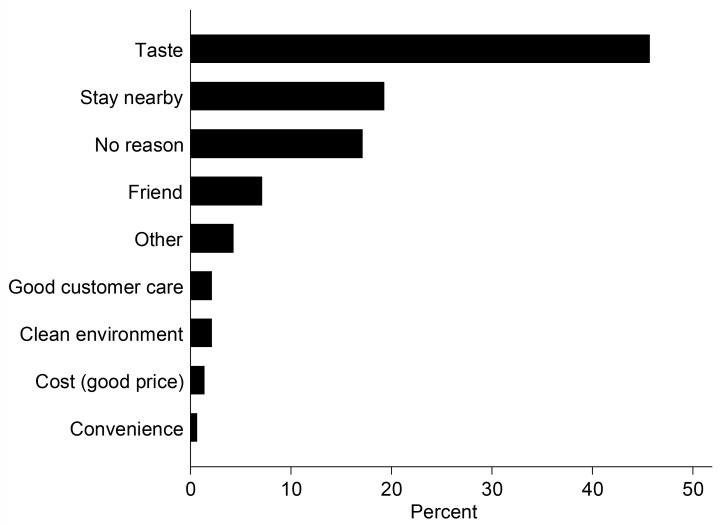
Main factors influencing street food consumers to buy prepared salad from vendors (N = 160).

**Fig 3:**
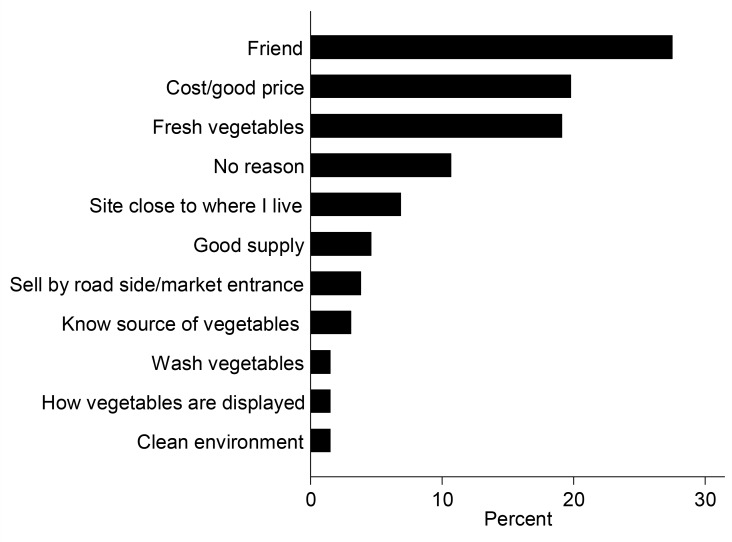
Main factors influencing domestic consumers to buy produce from market vendors (N = 160).

Despite the high awareness of health risk, street food consumers did not prioritise health indicators when buying food. Only 2% of consumers chose a vendor based on food safety reasons (Fig 2). Instead, consumers were more concerned about the taste of the food (46%), and the proximity of their house/work place to vending sites (19%). A higher number of consumers also seemed satisfied with sanitation conditions (82%), and how vendors prepared their salad (71%), despite the poor environmental and food hygiene practices at the vending sites. Similar to street food consumers, there was high variability in domestic consumers’ attitudes about indicators used to assess produce safety. Buyers’ choice of a vendor for produce was based primarily on friendship/good customer care (28%) and good price (20%) (Fig 3). Health indicators like clean environment, and how well produce had been displayed were lower priority for consumers (Fig 3). Domestic consumers of produce also seemed satisfied with how produce was displayed (82%), and the general sanitation at vending sites (62%). Market vendors in an FGD stated that they were most concerned about keeping their vegetables fresh, and ensuring environmental cleanliness in order to attract customers.

### Salad consumption patterns

The large majority of street food consumers (90%) reported to consume salad for an average of 4 times a week, while those (51%) who consumed salad at home did so for 2.8 times per week. Consumers of street food (“check-check”) attributed the taste/likeness of the food (49%) and convenience (30%) as the main motivator for their consumption; aside from cost (11%). Gender, occupation, age group and religion of consumers had no significant influence on their consumption of salad. After controlling for occupation, street food consumers, who were aware of wastewater related health risk were 2.2 times less likely to buy salads, or add salad to their food, if they knew the produce was wastewater irrigated (95% CI = 1.1–4.7, p = 0.03, Table 5).

**Table 5:** Determinants of buying wastewater irrigated produce used for salad among street food consumers (Univariable and Multivariable logistic regression models). OR^c^ = crude odds ratio, OR^a^ = adjusted odds ratio, NA = not applicable.

			Univariable Analysis		Multivariable analysis	
Exposure	N = 160	Buy (%)	OR^c^ (95% CI)	P*-value	OR^a^ (95% CI)	P^†^-value
Religion						
Muslim	100	45	1.0	0.38	NA	NA
Christian	58	38	0.7 (0.39, 1.45		NA	
Gender						
Male	96	35	1.0	0.03	1.0	0.71
Female	63	52	2.0 (1.05, 3.83)		1.2 (0.53, 2.53)	
Occupation						
Trading	57	47	1.0	0.01	1.0	0.01
Vocational	15	60.	1.7 (0.52, 5.30)		2.8 (0.55, 5.82)	
Student	36	53	1.2 (0.54, 2.86)		1.4 (0.59, 3.32)	
Scrap dealer	22	18	0.2 (0.07, 0.82)		0.2 (0.06, 0.75)	
Other	29	28	0.4 (0.16, 1.11)		0.4 (0.15, 1.11)	
Age group						
≤ 20	45	53	1.0	0.07	1.0	0.22
21–30	93	41	0.6 (0.30, 1.24)		0.76 (0.32, 1.81)	
> 30	21	24	0.3 (0.09, 0.87)		0.31 (0.08, 1.24)	
Awareness of wastewater health risk						
Yes	105	38	1.0	0.10	1.0	0.03
No	52	52	1.8 (0.90, 3.44)		2.2 (1.09, 4.66)	

*P-value from logistic regression.

^†^ p–value calculated from likelihood ratio test

Despite this observation, over 40% of street food consumers indicated that they would still consume salads even if the produce were wastewater irrigated (Fig 1). Street food consumers would often quote the Ghanaian proverb *“Ani ahu a*, *ɛnyɛtan”* to describe their reaction to the fact that farmers may be using wastewater for irrigation or vendors not preparing their salad in a hygienic way but *“If the eye does not see these things*, *it does not make the food disgusting”*. For domestic consumers, buying produce was not influenced by awareness of health risk, nor was it influenced by the source of produce (Table 6). Buying of produce was, however, strongly associated with knowledge of the source of irrigation water used for vegetable cultivation with those who were aware of the source of water 8 times less likely to buy wastewater irrigated produce (p < 0.001, Table 6).

**Table 6:** Determinants of buying wastewater irrigated produce at markets among domestic consumers of vegetables^†^ (Univariable logistic regression model).

Exposure	N = 160 (%)	Buy (%)	OR (95% CI)	P** - value
Religion				
Christian	133 (83)	19*	1.0	0.22
Muslim	27 (17)	30	1.8 (0.72, 4.63)	
Gender				
Male	25 (16)	16	1.0	0.52
Female	135 (84)	22	1.4 (0.46, 4.52)	
Occupation				
Trading	82 (51)	21	1.0	0.32
Government worker	17 (10.6)	0.0	-	
Vocational	49 (31)	23	1.1 (0.47, 2.61)	
Others	12 (7.5)	42	2.7 (0.77, 9.68)	
Age group				
≤ 30	57 (35.6)	25	1.0	0.55
31–40	49 (31)	23	1.1 (0.43, 2.74)	
40–50	37 (23)	14	0.6 (0.19, 1.83)	
> 50	17 (10.6)	29	1.6 (0.46, 5.30)	
Awareness of wastewater health risk				
No	32 (20)	19	1.0	0.83
Yes	127 (80)	21	1.1 (0.42, 2.99)	
Awareness of source of produce				
No	90 (57)	19	1.0	0.51
Yes	69 (43)	23	1.3 (0.60, 2.80)	
Awareness of source of irrigation water				
Yes	116 (73)	10	1.0	< 0.001
No	43 (27)	49	8.3 (3.55, 19.26)	

* Percentage of Christians who would buy wastewater irrigated produce

** p-value calculated from logistic regression

^**†**^No Multivariable model as only one parameter (source of irrigation water) was significantly associated with buying wastewater irrigated produce in the Univariable logistic model.

## Discussion

This study found that awareness of the source of irrigation water used for crop production was low among consumers and street food vendors, though higher among market vendors. Similarly, awareness of wastewater irrigation health risk was relatively low among farmers, but high among vendors and consumers, though this did not necessarily influence their decision to buy produce, or consume salad. The study further showed that awareness of health risk did not necessarily mean knowledge of the actual health risk, or source of risk, nor in the use of health protective practices among farmers, vendors and consumers.

### Awareness of the source of produce

In order to protect human health from the possible negative health impact of wastewater use in agriculture, the World Health Organisation has promoted a multiple-barrier approach. The success of this approach will depend largely on whether consumers know exactly where their produce originated from, and what it was irrigated with, but also whether they know what they can do to minimise health risks. In this study, awareness about where and what produce was irrigated with was reportedly low among consumers but higher among vendors of produce at local markets. The low awareness of the sources of irrigation water, or the source of produce was also corroborated by a review study that found that most buyers and consumers in Accra were actually unaware of the sources of produce, or the use of polluted irrigation water since they rarely ask these from vendors [8]. It is estimated that 50% to 90% of vegetables consumed by urban dwellers in West Africa are wastewater irrigated [22], which, inadvertently suggests that it might be inadequate to rely on the town, or country to determine the source of irrigation water, as was done by market vendors in the current study. It must also be noted that even though a significant proportion of market vendors, and chefs at restaurants and hotels reported to buy produce directly from farmers in Accra who often use wastewater for irrigation, the results of this study is unclear on the actual proportion of produce at markets and kitchens that was actually wastewater irrigated. It is also possible that not all the produce sold at these markets and kitchens are actually wastewater irrigated but with other sources of irrigation water. In addition, a study in Pakistan has shown that post- harvest contamination of produce occurs and could even pose a greater risk for consumers than the risk from wastewater [7]. Moreover, a farm-to-fork study which was part of a bigger study as the current study, also found prepared salad from street food vendors to be more contaminated than produce collected directly from wastewater irrigated fields, which also gives some credence to the significant impact of post-harvest contamination of produce [20]. The studies in Pakistan and Ghana have also suggested the need to prioritise risk reduction interventions at markets and kitchens as most buyers and consumers buy produce directly from these domains, and not at the farm level. This current study also found that vendors and consumers rarely considered the source of produce when buying produce but rather were influenced by other indicators particularly the freshness of the vegetables, confirming other findings in Ghana and in the United States [23, 24].

Urban agriculture is often promoted as a way to ensure a livelihood for the urban poor, and to grow fresh produce close to the places where it is sold to consumers [10, 25, 26]. The short distances to market also mean that transport and refrigeration cost are low, making the produce cheaper. This is a win-win situation for farmers, market vendors and consumers. Telling consumers where the produce originated from might turn some consumers away from buying this produce, as was shown by this study, as the use of domestic or municipal wastewater holds not only health risks, but is also a taboo in Ghana [11]. This will result in a loss of revenue, and making it unlikely that market and street food vendors will advertise the origin of their produce. However, for the multiple-barrier approach to function successfully, this is what is required. This is not a task for farmers, or market vendors, instead the involvement of local health, agricultural agencies and other key institutions is required, that can put practices in place that ensure crop safety but won’t deprive farmers and market salesmen of their livelihood; for example through making clean water available at agricultural fields and markets so that produce can be washed before it is sold.

### Health risks awareness among farmers

Farmers in this study reported the lowest levels of health risk awareness when it came to the use of wastewater in irrigation (37%), even though they should be the first ‘implementers’ of food safety measures when adopting a multiple barrier approach. Keraita *et al*. [11] and Qadir *et al*. [8] have reported similar findings from studies in Ghana attributing it to illiteracy, lack of adequate information and resources, and the fact that farmers have been exposed to poor sanitary conditions and unsafe water sources for most of their lives, and therefore tend to accept the risks in exchange for the benefits of their occupation. It was also possible that farmers in low and middle income countries may have developed some level of immunity or resilience to pathogens from wastewater, and hence their reported low awareness of health risk. This current study also showed that improved education was strongly associated with higher awareness of wastewater related health risk. Awareness of health risk associated to wastewater use was higher among wastewater farmers in Kenya (53%) and Bulawayo, Zimbabwe (70%), [13, 27]. In Pakistan, farmers irrigating with untreated wastewater stated that there were no negative health impacts associated with wastewater use [28], and there, as well as in Ghana this was contributed to a defensive mechanism to protect their livelihood, and not necessarily a lack of knowledge of health risk, or protective measures [11]. Farmers are often acutely aware that the use of wastewater for vegetable cultivation is illegal and frowned upon by society, and therefore in order to keep their job; tend to underreport the associated health risk or prevalence of wastewater use, or in some cases argue that their source of irrigation water poses no risk [11]. However, farmers are willing to adopt risk reduction measures to avoid further pressure from city authorities and the media, as a study in Accra found [11], though land insecurity has been suggested as one of the key reasons for farmers not investing in measures that could reduce health risks, like drip irrigation, use of boreholes and on-farm sedimentation ponds [10, 29].

### Adoption of protective measures

Several reasons have been suggested for the low adoption of health protective measures when buying produce, or prepared food, which included low awareness of the sources of irrigation water and a much lower priority of health parameters, including the hygiene conditions at food vending sites. The study found that although awareness of health risk related to wastewater use in agriculture was high (67%–80%), it did not necessarily translate into the adoption, or usage of health protective measures or health indicators when buying food (2%–25%).

The fact that risk awareness, and knowledge of risks, do not translate into healthy behaviour has been shown in past hygiene programmes; with several studies having shown that the presence of soap in household and knowledge of when to wash hands, did not translate in high hand washing rates at key times [30, 31]. A study in Ghana found high education levels among mothers (73%), and 55% knew of at least two of the three key times when to wash hands with soap, though the prevalence of hand washing with soap was very low (3.6%) [30]. Biran *et al*. [32] have also speculated that efforts to change hand washing behaviours at large scale have achieved little success because they relied on communicating health benefits of hand washing with soap, rather than giving more attention to the effect of emotional drivers.

Our results also show that being aware of the health risk did not necessarily influence the buying of produce (OR = 1.1) particularly among domestic consumers, though it had significant influence on street food consumers’ decision to consume street food salad. The first possible explanation was that most salespersons and consumers were unaware of the sources of produce, or the quality of irrigation water, confirmed by the fact that those that were aware, were less likely to buy wastewater irrigated produce. However, most vendors and consumers (> 90%) were motivated to buy produce, or consume salads using indicators unrelated to food hygiene and safety, like friendliness of the vendor, and taste. A further reason stated was a lack of time or money. These findings are similar to those from central Ghana (Kumasi) where consumers relied mainly on neatness, appearance and trustworthiness of the vendor, in addition to cost and accessibility in their choice for a food vendor [33]. Interestingly, both neatness and trust were construed by vendors and consumers using indicators of different dimensions to the normal classical definition of these words [33]. Convenience, or a lack of time is an important driving force to buy produce at one particular place, or to use food vendors where the food quality is unknown. In addition, friendship relates to social networks, and studies have found a strong association between social networks and communication among, within and between people and the adoption of new behaviours (e.g. food consumption) which could also lead to improved health benefits and reduction of health risks or exposures [34–36].

The findings of this study suggest that relying on health indicators to raise awareness or promote the uptake of health protective measures may not be sufficient to influence positive behaviour change. Campaigners of hand washing promotion programmes and the community led total sanitation (CLTS) approaches have suggested that the use of emotional drivers like disgust, habit formation, nurture and affiliation could better engender long lasting behaviour change than the promotion of health benefits which could only be effective in cases of disease epidemics [32, 37, 38]. The use of emotional drivers could also be particularly important in situations where the severity or consequences (e.g. death or serious sequelae) of the health risk is unknown, or cannot be determined by the at risk groups.

Aside from emotional drivers, the promotion of interventions that would create more direct benefits to producers, vendors and consumers must be prioritised. These interventions are likely to be successful if they are implemented using participatory approaches that build upon existing practices of farmers and vendors and should require minimal changes to current practices and low capital investment from farmers and salespersons [11]. For example, farmers in this study felt a lack of support from government in respect to agricultural extension support. Agricultural extension programmes could also incorporate food hygiene, and advice on how to minimize negative health impact to consumers and farmers, and possibly provide support to install clean water points, where produce can be washed. In this study, the weak relationship between farmers’ associations and local authorities could also be due to insecure land tenure arrangements for farmers, and a lack of representation and communication. In Kenya, a study found that farmers with recognised membership, and those with access to certified seed and credit were more likely to adopt innovative risk reduction interventions to minimize health risks linked to wastewater irrigation [13]. This study did not assess the efficacy and feasibility of risk reduction measures at the farm level, and hence it is unclear how practicable and effective the WHO recommended measures in the multiple-barrier approach could be implemented even if farmers were supported to do so. Future studies should consider these factors in order to confirm the practicalities of the WHO risk reduction measures at the farm level.

Similar to interventions to increase the uptake of risk reduction measures at the farm level, collaborative approaches and a conducive environment are needed to increase trust between vendors and local authorities, the media and consumers regarding food safety. One way of handling this is to develop effective communication channels between farmers, vendors and consumers on risk reduction measures based on the multi-barrier approach. Local authorities should also require vendors to join vendor associations in order to benefit from hygiene and behaviour change education programs. Currently, most market and street food vendors have no experience in hygiene training but the award of training certificates on food hygiene and safety could increase consumer confidence, and trust in vendors, which possibly could benefit vendors with increased sales [20]. Consumers have a significant influence on vendors and farmers, and can contribute to the adoption of safer practices by buying from only those who adhere to hygienic requirements. Market vendors can also capitalise on societal pressure on farmers for safer food by branding safer production sites with names associated with accepted norms such as ‘neat’ and ‘clean’ similar to market vendors’ supposed preference of carrots from Togo than those from Ghana [39].

## Conclusion

The multiple-barrier approach is a key component of the WHO guidelines but can only function effectively if relevant stakeholders are committed to play their roles of ensuring public health safety. For farmers and salespersons, and also for consumers, the adoption of this approach may be influenced in part by the recognition of their vulnerability to health risk from wastewater, or wastewater irrigated produce, and what risk avoidance methods they need to practice to protect themselves; but more importantly the direct benefits they will get by adhering to the approach. The results from this study showed that even when people were aware of the health risk associated with wastewater irrigation, or were aware of the source of produce, it was not sufficient to influence their adoption of health protective measures, or influence whether they buy and consume wastewater irrigated produce. However, people were likely to change behaviours, including the adoption of risk reduction measures if they knew the actual source, or type of irrigation water used for vegetable production. The study findings also seem to suggest that consumers of salad vegetables were less concerned, or rarely relied on health indicators in their choice for vendors, and subsequently in their decision to buy produce or consume salad.

From the foregoing, it is clear that in order to reduce health risks, interventions that could more directly impact benefits (especially economic benefits) to producers, salespersons and consumers of salad crops should be promoted, rather than relying on health promotion and awareness. These interventions could include credit scheme support, and also the award of safety certificates to farmers and vendors who comply with prescribed risk reduction measures including good agricultural practices at farms and hygienic practices at markets and kitchens. Above all, interventions are likely to be successful if they are implemented in a participatory manner to involve government, at-risk groups and other major stakeholders.

## Supporting Information

S1 Fig Map of Urban Agriculture sites in study communities in Accra. (PDF)

S2 Fig Map of Ghana showing study sites in Accra. (PDF)

S1 Appendix Data collection tools. (PDF)
