# Supplementary figures and images for: Risk Perceptions of Wastewater Use for Urban Agriculture in Accra, Ghana
Source: PLoS One. 2016 Mar 15;11(3):e0150603. doi: 10.1371/journal.pone.0150603 (PMC4792467; doi:10.1371/journal.pone.0150603)

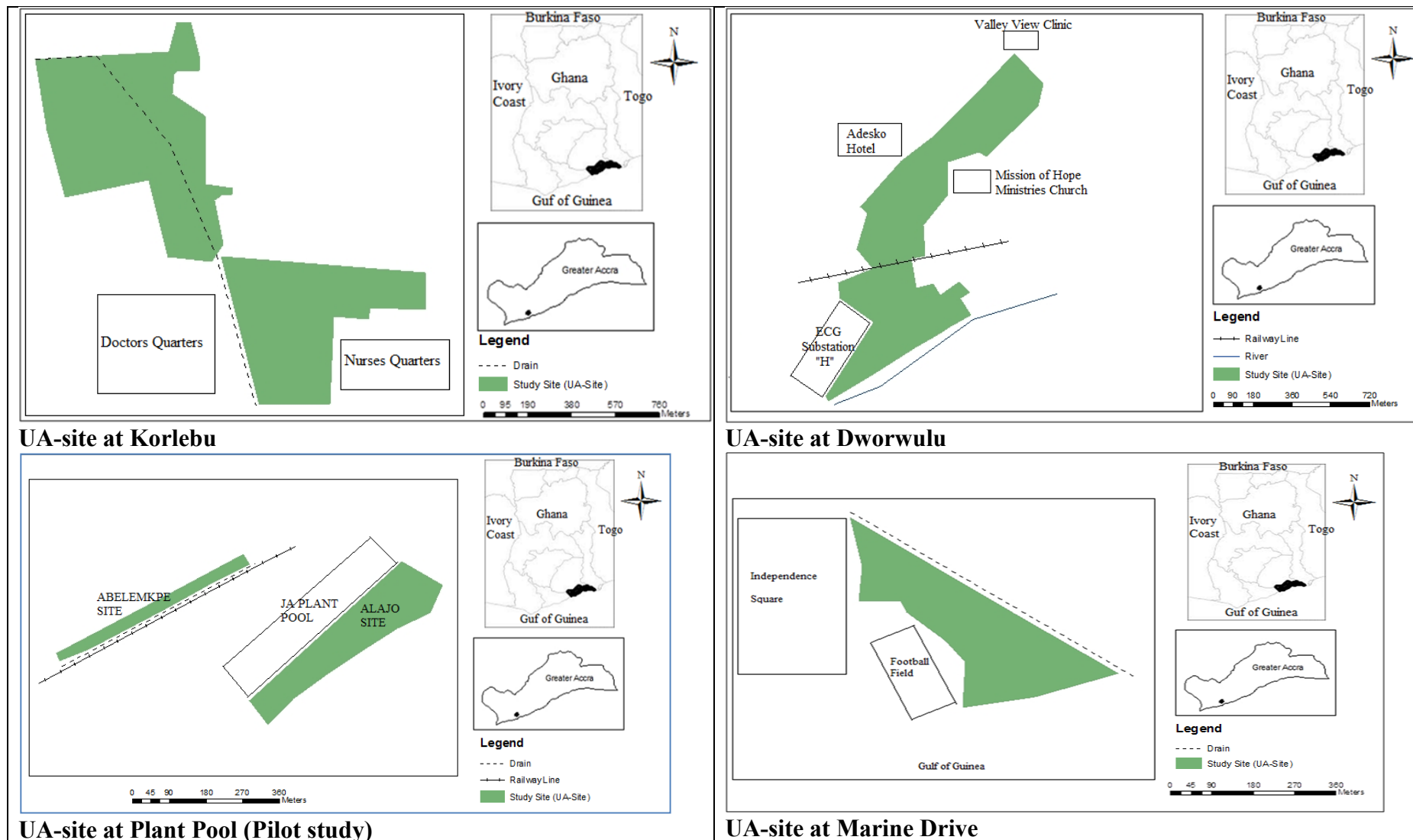

S1 Fig. Map of Urban Agriculture sites in study communities in Accra

Supplement: S1 Fig — (PDF) [file pone.0150603.s001.pdf]
