# Supplementary material for: Risk Perceptions of Wastewater Use for Urban Agriculture in Accra, Ghana
Source: PLoS One. 2016 Mar 15;11(3):e0150603. doi: 10.1371/journal.pone.0150603 (PMC4792467; doi:10.1371/journal.pone.0150603)
Supplement: S2 Fig — (PDF) [file pone.0150603.s002.pdf]

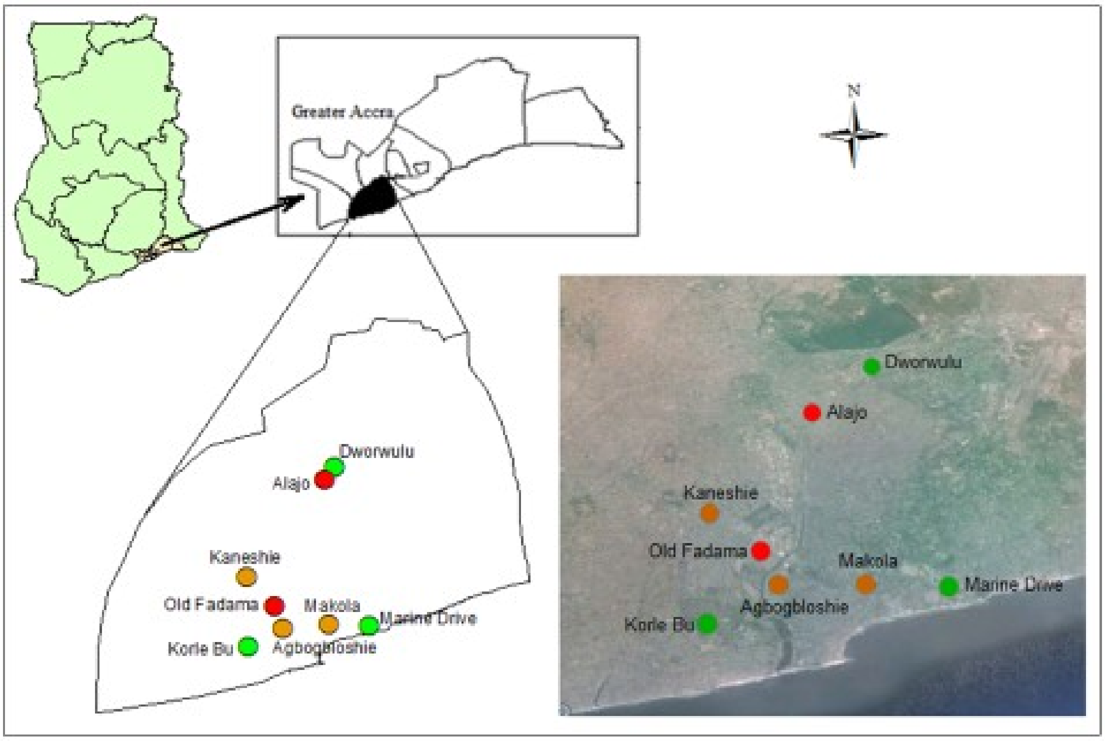

- *Wastewater irrigated fields,* -*Markets,* - *Street food vending sites*

S2 Fig. Map of Ghana showing study sites in Accra
